# Analysis of and prognostic information from disseminated tumour cells in bone marrow in primary breast cancer: a prospective observational study

**DOI:** 10.1186/1471-2407-12-403

**Published:** 2012-09-11

**Authors:** Anna-Karin Falck, Pär-Ola Bendahl, Christian Ingvar, Jorma Isola, Per-Ebbe Jönsson, Pia Lindblom, Kristina Lövgren, Karin Rennstam, Mårten Fernö, Lisa Rydén

**Affiliations:** 1 Department of Surgery, Clinical Sciences, Lund University, Lund, SE-22185, Sweden; 2 Department of Surgery, Hospital of Helsingborg, Helsingborg, SE-25187, Sweden; 3 Department of Oncology, Clinical Sciences, Lund University, Lund, SE-22185, Sweden; 4 Department of Surgery, Skåne University Hospital, SE-22185, Lund, Sweden; 5 Institute of Medical Technology, University of Tampere, Tampere, FI-33014, Finland

**Keywords:** Breast cancer, Disseminated tumour cells, Cytokeratin-positive cells, Micrometastases, Prognosis

## Abstract

**Background:**

Disseminated tumour cells (DTCs) in the bone marrow of patients with breast cancer have been identified as an independent predictor of poor prognosis in patients with non-metastatic disease. This prospective study aimed to evaluate the presence and prognostic value of DTCs in the bone marrow of female patients with primary breast cancer.

**Methods:**

Between 1999 and 2003, bone marrow aspirates were obtained from patients at the time of surgery for primary invasive breast cancer. DTCs in bone marrow were identified using monoclonal antibodies against cytokeratins for detection of epithelial cells. The detection of DTCs was related to clinical follow-up with distant disease-free survival (DDFS) and breast cancer-specific survival as endpoints. Bone marrow aspirates from adult healthy bone marrow donors were analysed separately.

**Results:**

DTCs were analysed in 401 patients, and cytokeratin-positive cells were found in 152 of these (38%). An immunofluorescence (IF) staining procedure was used in 327 patients, and immunocytochemistry (IC) was performed in 74 patients. The IF-based method resulted in 40% DTC-positive cases, whereas 30% were positive using IC (p = 0.11). The presence of DTCs in bone marrow was not significantly related to patient or tumour characteristics. The presence of DTCs was not a prognostic factor for DDFS (IF: hazards ratio [HR], 2.2; 95% confidence interval [CI], 0.63–2.2; p = 0.60; IC: HR, 0.84; 95% CI, 0.09–8.1; p = 0.88). Significant prognostic factors were lymph node metastases, oestrogen receptor positivity, Nottingham histological grade, and tumour size using Cox univariate analysis. The analyses were positive for epithelial cells in bone marrow from adult healthy donors in 19 (25%) samples.

**Conclusions:**

The detection of DTCs in bone marrow in primary breast cancer was previously shown to be a predictor of poor prognosis. We were not able to confirm these results in a prospective cohort including unselected patients before the standard procedure was established. Future studies with a standardised patient protocol and improved technique for isolating and detecting DTCs may reveal the clinical applications of DTC detection in patients with micrometastases in the bone marrow.

## Background

Breast cancer remains the most common cancer diagnosis among women in Sweden today, with an incidence of 7400 patients per year. It is generally associated with a good prognosis; more than 85% of Swedish patients are free from recurrence of the disease at the 5-year follow-up because of early detection combined with extended adjuvant therapy [1].

Adjuvant treatment is delivered to eradicate micrometastatic spread at the time of diagnosis and thus minimise the risk of subsequent clinically overt metastasis from the micrometastatic stage. Adjuvant treatment is tailored to a prognostic profile including validated prognostic factors (age, nodal status, tumour size, Nottingham histological grade [NHG], and human epidermal growth factor receptor 2 [HER2] amplification) and predictive factors (hormone receptor status, HER2) [2]. Metastatic lymph node involvement (N+) is still considered to have the strongest impact of all accepted prognostic factors. However, approximately 30% of patients with no sign of metastatic involvement of the lymph nodes (N0) relapse and suffer from metastatic disease [3,4]. In contrast, 40% of N+ patients survive 10 years or more without recurrence [5,6]. The heterogeneity of breast cancer challenges the search for further prognostic markers that will provide a more direct measure of the disease’s metastatic potential. Recognition and understanding of the metastatic process includes investigation of the molecular mechanism of early spread of tumour cells.

Micrometastatic spread to bone marrow by disseminated tumour cells (DTCs), defined as cytokeratin (CK)-positive cells, occurs in up to 40% of patients with primary breast cancer at the time of diagnosis [7-10]. DTCs seem to be unrelated to lymphatic spread and occur in both N0 and N+ disease [7-9], and no distinct pattern is found in relation to standard prognostic factors [8,9,11,12]. The prognostic influence of DTCs in bone marrow has been evaluated by several groups over the last 30 years with the aim of finding a tool to detect micrometastatic disease at the time of diagnosis [10,13,14]. There has been an increasing acceptance of DTCs as an independent marker of a poor prognosis in breast cancer after the publication of a pooled analysis [7], and DTCs are now included in the new American Joint Committee on Cancer classification as a diagnostic criteria for micrometastatic spread. Early detection of these epithelial cells may help to identify patients with micrometastatic disease who would benefit from adjuvant treatment to prevent further metastatic disease. However, aspiration and analysis of bone marrow for detection of DTCs as a prognostic tool is not yet a routine clinical procedure [15] and is not recommended by the American Society of Clinical Oncology [16]. Aspiration may be associated with pain and discomfort for the patient, particularly if performed repeatedly to monitor treatment. Furthermore, the increasing acceptance of DTCs as a prognostic factor is based on studies using different CKs as well as membrane antibodies to detect epithelial cells [7,15]. Comparisons among different detection methods were performed [17] before standardised guidelines were published [15]. These comparisons showed difficulties in interpreting CK-positive cells as tumour cells and recommended the use of markers that allow discrimination between CK-positive cells of haematopoietic and non-haematopoietic origin. To ensure validation of the method used, it is important to have tumour cell samples as positive controls, specific negative controls, and bone marrow samples from healthy individuals. Results from clinical studies will vary until the technique has been standardised and the optimal dilution with antibodies has been identified.

The aim of the present study was to detect the presence of DTCs and analyse the prognostic implications of DTCs in bone marrow at the time of diagnosis in a prospective cohort of patients with primary breast cancer. An additional aim was to further stratify the cohort according to lymph node status to enable the clinical information obtained in N+ and N0 patients to be studied separately.

## Methods

### Patients

This study included patients (median age, 58 years) diagnosed with primary breast cancer in the South Swedish Health Care Region between June 1999 and May 2003 as well as patients diagnosed in Lund, Landskrona and Helsingborg. The patients underwent bone marrow aspiration from the sternal crest under anaesthesia at the time of primary surgery. The study was approved by the ethics committee at Lund University, and written informed consent was obtained from all included patients (LU699-09, LU75-02).

Patient and tumour characteristics are given in Table 1. Patients underwent either mastectomy or breast-conserving therapy based on preoperatively identified characteristics and staging. A sentinel node biopsy was performed in patients with no sign of axillary node engagement before surgery, followed by axillary lymph node dissection (level I and II) at the time of either the primary operation or a second operation if histopathological analysis showed metastatic involvement in the sentinel node biopsy. If node involvement was known preoperatively, axillary lymph node dissection was performed at the time of the primary surgery.

**Table 1:** Patient- and tumor characteristics

**Characteristics**	**No of patients (%)**
All patients	401 (100)
**Age**	
Median (range)	58 (29–91)
< 50 years	80 (20)
≥ 50 years	321 (80)
**Mode of detection**	
Screening detected	167 (42)
Clinical signs	232 (58)
Unknown	2
**Tumor size**	
≤ 20mm	263 (66)
> 20mm	136 (34)
Unknown	2
**Node status**	
N0	233 (60)
N+	157 (40)
Unknown	11
**NHG**	
1	77 (20)
2	221 (56)
3	94 (24)
Unknown	9
**ER status**	
Positive	312 (80)
Negative	77 (20)
Unknown	12
**PR status**	
Positive	242 (62)
Negative	147 (38)
Unknown	12
**Surgery breast**	
Mastectomy	157 (39)
Breast concerving surgery	243 (61)
Unknown	1
**Surgery axilla**	
Axillary dissection	247 (63)
Sentinel node biopsy	142 (37)
No Surgery (incl in clinical trial)	12
**Adjuvant treatment** No adjuvant treatment	137 (34)
Only Chemotherapy	65 (16)
Only Endocrine therapy	197 (49)
Missing	2
**Radiotherapy**	
Breast	198 (49)
Locoregional lymph nodes	35 (9)
Breast + locoregional	39 (10)
	

Abbreviations: N0= node negative, N+= node positive, NHG= Nottingham histological grade, ER= oestrogen receptor, PR= progesterone receptor.

Adjuvant therapy was recommended according to clinical standards following Regional Guidelines, and included chemotherapy for N+ premenopausal women and N+ postmenopausal women with oestrogen receptor-negative tumours (ER-) (n = 65, 16%). Endocrine therapy was delivered to 197 patients (49%); premenopausal patients with oestrogen receptor-positive (ER+) tumours and no nodal engagement received tamoxifen, and postmenopausal women with ER + tumours received tamoxifen or aromatase inhibitors regardless of nodal status. Chemoendocrine therapy was given to 22 patients; 137 patients had no adjuvant therapy. Radiotherapy to the breast was given after breast-conserving surgery (50 Gy) (n = 198, 49%) and locoregional radiotherapy was delivered if four or more axillary lymph nodes were metastatic (n = 35, 9%). A combination of radiotherapy to the breast and to the locoregional lymph nodes was delivered in 39 patients (10%).

The patients were followed by annual clinical examination and mammography. Further clinical and radiological examinations were performed when clinical signs indicated recurrence of the disease. After 5 years of follow-up, all clinical and histopathological results concerning tumour grading and staging, as well as reports of events, were abstracted from individual patient’s charts. The median follow-up for patients without any breast cancer-related event was 61 months. For patients for whom no cause of death was registered, we received information from the Swedish Register of Causes of Death (Central Statistics Office). The inclusion criteria were re-evaluated.

The original cohort included 569 patients, 544 of whom were followed according to the schedule. The exclusion criteria were no standardised surgical treatment (laser, n = 1), neoadjuvant treatment (n = 11), and local recurrence (n = 3) and the sample volume was inadequate in 36 patients. The analysis was not performed in 117 patients due to change in research strategy at our laboratory. The final cohort thus included 401 patients.

### Bone marrow

Bone marrow aspirates were obtained from the sternum at two sites by needle aspiration while the patient was under general anaesthesia at the time of primary surgery. The samples were transported to the research laboratory at room temperature and prepared within 1 h. Mononuclear cells were separated by Ficoll density gradient centrifugation (Ficoll-Paque™ PLUS, Cat. no. 17-1440-03; Amersham Biosciences AB, Uppsala, Sweden) and then washed and counted before 1.5 to 2.0 × 10^6^ cells were placed on each glass slide. Two microscope slides were prepared for each patient, one from each site at the sternum.

## Immunofluorescence and immunocytochemical analysis

In 327 patients, an immunofluorescence (IF) staining procedure was used for detection of DTCs, including staining with antibodies against CKs (Pan-CK Ab-2; Neomarkers, Union City, CA, USA) 4, 5, 6, 7, 8, 10, 13, 14, 15, 16, 18, and 19 and visualised by IF using an IF microscope (Axioplan 2; Zeiss, Jena, Germany). The cytospins were incubated with the pan-CK antibody and a secondary fluorescein isothiocyanate-conjugated goat antimouse antibody (Zymed Laboratories Inc., San Francisco, CA, USA) and finally counterstained with 4,6-diamidino-2-phenylindol in mounting media using Vectashield (Vector Laboratories, Burlingame, CA, USA). The procedure changed when a new CK antibody kit (AE1/AE3; Daco, Glostrup, Denmark) was introduced with antibodies against CKs 1, 2, 3, 4, 5, 6, 7, 8, 10, 13, 14, 15, 16, and 19. For the IC method, the cells were fixed in buffered formaldehyde (4%) and thereafter pre-treated in citrate buffer (pH 6) in a microwave oven for 20 minutes. The cytokeratin antibody kit (AE1/AE3, Daco, Glostrup, Denmark) was used as primary antibodies against CK1,2,3,4,5,6,7,8,10,13,14,15,16 and 19. The EnVision™ (Dako, Glostrup, Denmark) was used as detection system, NovaRed™ (Vector Laboratories, Immunkemi AB, Sollentuna Sweden) for the visualisation and Mayers hematoxylin for nuclear staining. This enables direct immunocytochemical evaluation (IC) of the cells and analysis by light microscope (Olympus CX41, Tokyo, Japan) in 74 patients.

**Figure 1:**
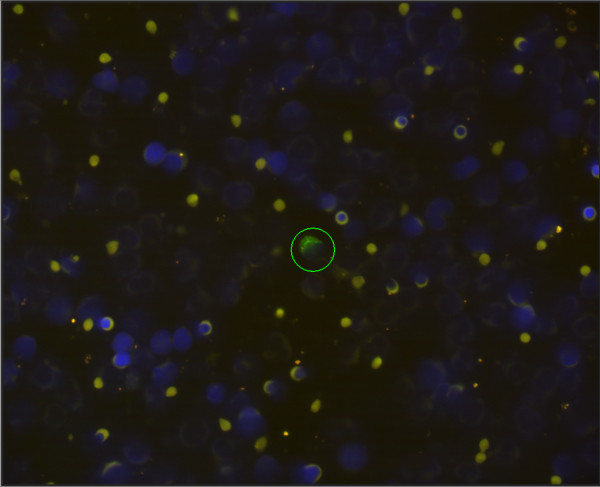
Cytokeratin staining of DTCs isolated from bone marrow by immunofluorescence.

**Figure 2:**
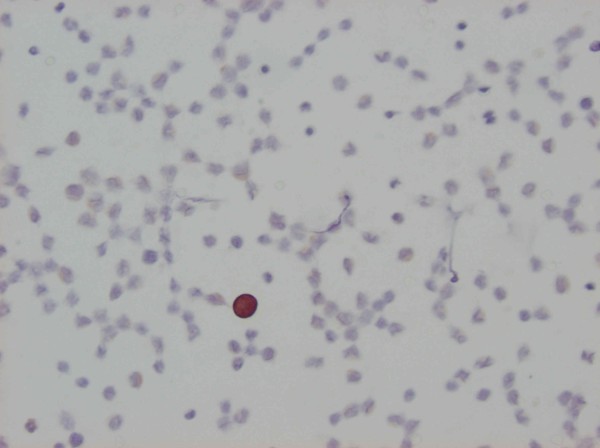
Cytokeratin staining of DTCs isolated from bone marrow by immunocytochemistry.

The presence of DTCs was defined as CK-positive cells with DTC morphology (irregular staining of the cytoplasm) with an enlarged nucleus, irregularity of the nucleus, a high nuclear-to-cytoplasmic ratio, CK staining of the cytoplasm at the periphery of the cell causing a ring-like appearance, and fluorescence-positive intact cells (IF technique) according to Fehm [8]. For the IC evaluation, we followed the criteria proposed by Borgen, who used the same antibody [18,19]. The criteria include moderate to strong staining intensity for the entire cytoplasm in mononuclear cells lacking haematopoietic characteristics [18,19]. The evaluation was performed by two observers independently. All specimens were considered either positive or negative when one or more CK-positive cell was diagnosed. DTCs detected by IF are illustrated in Figure 1 and by IC in Figure 2.

As a positive control for CK immunostaining, we used the breast cancer cell line MCF7 spiked into blood from healthy volunteers. The cell line was kindly provided by Prof. Stina Oredsson at Lund University. The slides for negative controls were prepared in parallel with those stained with anti-CK by omitting the primary antibody, and thus contained the same number of cells (1.5 to 2 × 10^6^). No positive results were observed in the negative controls.

Bone marrow aspirates from 76 adult healthy bone marrow donors were analysed separately.

### Statistical analysis

Distant disease-free survival (DDFS) and breast cancer-specific survival (BCSS), measured from the month of surgery to the last clinical follow-up or any breast cancer-related event, were used as endpoints. DDFS included distant metastases to the skeleton, brain, lung, and liver.

Differences in the distribution of clinical data and tumour characteristics between the DTC+ and DTC- patients were evaluated using the *X*^2^ test. DDFS and BCSS were estimated according to the Kaplan-Meier method, and the log-rank test was used to compare survival in different subgroups. The Cox proportional hazards model was fitted to explore the effects of tumour size, lymph node status, ER and progesterone receptor content, age, and NHG on BCSS and DDFS. Proportional hazards assumptions were checked graphically.

P-values of <0.05 were considered statistically significant. The statistical software package Stata 11.1 (Stata Corp., College Station, TX, USA) was used for all statistical calculations.

## Results

### Detection of micrometastases

Analysis of DTCs was performed in 401 patients, and CK-positive cells were found in 152 of these (38%). The IF-based method resulted in 40% DTC-positive cases, whereas 30% were positive using the IC method. However, there was no statistically significant difference between the detection rates of the two methods (p = 0.11). The number of positive cells was not taken into account.

### Characteristics of DTCs and patients

Patient and tumour characteristics are listed in Table 1. The relationship between DTC detection in bone marrow and clinicopathological variables in the study cohort is presented in Table 2. There was no statistically significant correlation between the presence of DTCs and the characteristics, regardless of the method used for DTC detection.

**Table 2:** Patient`s and tumor characteristics in relation to presence of DTC in bone marrow

**Characteristics**	**Patients analyzed by IF**	**No DTC in bone marrow N (%)**	**DTC in bone marrow N (%)**	p-value*	**Patients analyzed by IC**	**No DTC in bone marrow N (%)**	**DTC in bone marrow N (%)**	p-value*
**Tumor size**								
≤ 20 mm	203	118 (58)	85 (42)	0.3	60	42 (70)	18 (30)	1.0
> 20 mm	123	78 (63)	45 (37)		13	9 (69)	4 (31)	
**Node status**								
N0	184	113 (61)	71 (39)	0.5	49	35 (71)	14 (29)	0.7
N+	133	77 (58)	56 (42)		24	16 (67)	8 (33)	
**NHG**								
1	60	30 (50)	30 (50)		17	13 (76)	4 (24)	
2	181	110 (61)	71 (39)	0.12	40	24 (60)	16 (40)	0.7
3	80	51 (64)	29 (36)		14	12 (86)	2 (14)	
**ER status**								
Positive	252	145 (58)	107 (42)	0.12	60	41 (68)	19 (32)	0.8
Negative	66	45 (68)	21 (32)		11	8 (73)	3 (27)	
**PR status**								
Positive	189	110 (58)	79 (42)	0.5	53	37 (70)	16 (30)	0.8
Negative	129	80 (62)	49 (38)		18	12 (67)	6 (33)	

* *X*^2^-test for two categories and *X*^2^-test for trend for three ordered categories.

Abbreviations: IF= immunofluorescence, IC=immunocytochemistry, DTC= disseminated tumor cells, N0= node negative, N+=node positive, NHG= Nottingham histological grade, ER= oestrogen receptor, PR= progesterone receptor.

**Figure 3:**
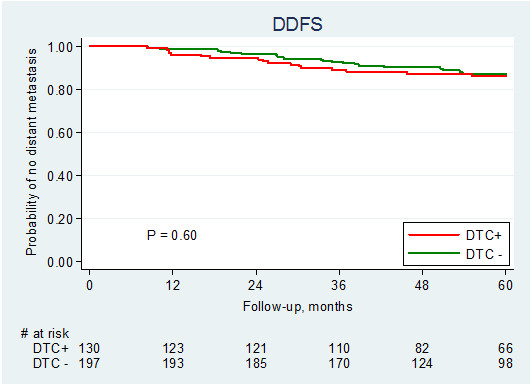
Distant disease-free survival (DDFS) in relation to presence of DTC.

**Figure 4:**
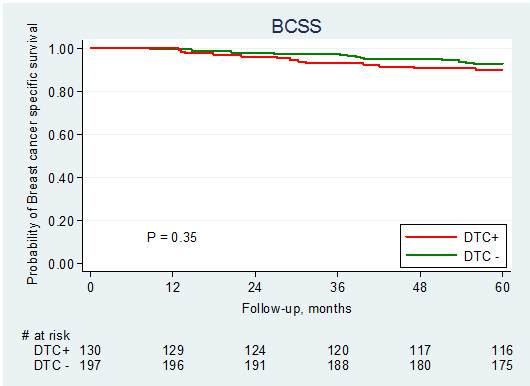
Breast cancer-specific survival (BCSS) in relation to presence of DTC.

For survival analysis, we initially included all 401 patients for whom DTC analysis was performed. In the cohort of patients analysed with the IF staining procedure, the detection of DTCs in bone marrow was not related to either DDFS (log-rank test, p = 0.60) (Figure 3) or BCSS (log-rank test, p = 0.37) (Figure 4). Stratifying the cohort according to the method used for the detection of DTCs resulted in similar results using Cox univariate analysis (Table 3). In Cox univariate analysis of DDFS, the following clinicopathological variables were related to prognostic information: lymph node metastases (+ *vs.* -: hazard ratio [HR], 5.5; 95% confidence interval [CI], 2.7–11), tumour size (>20 *vs.* ≤20 mm: HR, 4.9; 95% CI, 2.6–9.4), NHG (3 *vs.* 1: HR, 20; 95% CI, 2.7–147), ER (+ *vs.* -: HR, 0.39; 95% CI, 0.21–0.72), and PR, progesterone receptor (+ *vs.* -: HR, 0.43; 95% CI, 0.24–0.79). In a Cox proportional hazards model for DDFS, lymph node metastases (+ *vs.* -: HR, 3.6; 95% CI, 1.7–7.4), tumour size (>20 *vs.* ≤20 mm: HR, 2.5; 95% CI, 1.1–5.1), and NHG (3 *vs.* 1: HR, 8.7; 95% CI, 1.1–69) remained independent prognostic factors (Table 3). The results for BCSS were similar (data not shown).

**Table 3:** Cox univariate and multivariate analysis of distant disease-free survival

	**Univariate analysis (n ≤ 401)**			**Multivariate analysis (n = 377)**		
**Variable**	HR*	**95% CI**	**p-value**	HR**	**95% CI**	**p-value**
**DTC status (IF and IC)** (n=401) DTC+ *vs* DTC-	1.2	0.66-2.2	0.55			
**DTC status (IF)** (n=327)	1.2	0.63- 2.2	0.60			
DTC+ *vs* DTC-						
**DTC statuc (IC)** (n=74) DTC+ *vs* DTC-	0.84	0.09-8.1	0.88			
**Age**	0.99	0.97-1.02	0.61			
per year						
**Node status**	5.5	2.7- 11	< 0.001	3.4	1.6- 7.2	0.001
N+ *vs* N0						
**Tumor size**	4.9	2.6-9.4	< 0.001	2.5	1.2- 5.2	0.01
> 20 mm *vs* ≤ 20 mm						
**NHG status**	6.9	0.92-52	0.06	4.9	0.65-37	0.12
NHG 2 *vs* NHG 1						
NHG 3 *vs* NHG 1	20	2.7- 147	0.004	8.7	1.1- 70	0.04
**ER status**	0.39	0.21-0.72	0.003	0.85	0.38- 1.9	0.7
ER+ *vs* ER-						
**PR status**	0.43	0.24- 0.79	0.007	0.67	0.33- 1.4	0.3
PR+ *vs* PR**-**						

* No significant deviations from proportional hazards (Schoenfeld’s test).

* *P=0.05 in Schoenfeld’s global six degree-of-freedom test of proportional hazards.

Abbreviations: IF= immunofluorescence, IC=immunocytochemistry, HR= hazard ratio, CI= confidence interval, DTC= disseminated tumor cells, N0= node negative, N+= node positive, NHG= Nottingham histological grade, ER= oestrogen receptor, PR= progesterone receptor.

### Subgroup analysis

When the cohort was stratified according to lymph node status, Cox univariate analysis of N0 patients showed that the presence of DTCs had no statistically significant effect on prognosis in terms of DDFS (DTC+ *vs.* DTC-: HR, 2.7; 95% CI, 0.72–9.1; p = 0.14). In the N+ group of patients, the presence of DTCs had no significant effect on DDFS (DTC+ *vs.* DTC-: HR, 0.84; 95% CI, 0.42–1.72; p = 0.6). Although the presence of DTCs seemed to have a more pronounced effect in the N0 subgroup, the interaction between lymph node status and the presence of DTCs was not significant (p = 0.13). The results were similar in the subgroups of patients in whom DTCs were detected by IF and IC (data not shown).

The bone marrow from healthy adult bone marrow donors was analysed using both methods. The analyses were positive for epithelial cells in bone marrow in 19 (25%) samples, negative in 53 (70%), and inadequate or ambiguous in 4 (5%).

## Discussion

In the present study, the detection of DTCs in bone marrow in female patients with primary breast cancer at the time of diagnosis had no prognostic impact. Although most publications report that detection of DTCs in primary breast cancer is an independent prognostic factor for recurrence and death, the clinical significance of micrometastases in bone marrow remains controversial. The American Society of Clinical Oncology did not advocate it as a prognostic marker for clinical use because of insufficient data [16], and several concerns have been raised regarding the standardisation of detection using monoclonal antibodies against CKs. The standardisation of the detection method is based on IC using a strict protocol for negative controls and morphological evaluation of stained mononuclear cells. The present study included patients before the standard protocol was published [15], and the data are mainly derived from detection by an IF staining procedure that was not included in the published meta-analysis and is not advocated by the consortium [7,15].

The detection of DTCs in bone marrow has been identified in several publications as an independent predictor of poor outcome in patients with non-metastatic breast cancer disease [14,20,21]. The level of evidence increased when a pooled analysis of 4703 patients with breast cancer was published, assessing the poor prognostic significance of the presence of DTCs in the bone marrow at the 10-year follow-up [7]. The pooled analysis, which included a large patient cohort, also enabled the analysis of subgroups with statistical power. Interestingly, the largest difference in outcome for patients with DTC+ *vs.* DTC- disease was in the subgroup in which all patients received adjuvant systemic therapy. Although there was a significantly higher risk of relapse in patients with DTC+ disease compared with patients with DTC- disease in the subset of patients who did not receive adjuvant systemic therapy (n = 1036, 22%), the effect was relatively small, but still significant (5-year follow-up: incidence ratio, 2.0; 95% CI, 1.2–3.4). Aspects to consider when estimating the results of these early reports are the heterogeneity of the patients included and different methods and techniques used to determine bone marrow dissemination. However, more recent studies performed with standardised methods of detection also propose a prognostic value of DTCs in bone marrow [22-24]. Molloy et al. found clinical significance of DTCs in bone marrow in terms of BCSS (HR, 2.1; p = 0.003), but not in metastasis-free survival (HR, 1.5; p = 0.127) [23]. Giluiano et al. reported that DTCs were present in 104/3413 (3.0%) patients and was associated with decreased overall survival in univariate analysis, but this did not reach clinical validity in multivariate analysis. They concluded that bone marrow aspiration was not recommended in routine clinical practice for patients with early breast cancer without an improved technique for isolation and detection of occult tumour cells in bone marrow [22]. Solá et al. found a higher frequency of DTCs in a subgroup of patients who experienced breast cancer-related events (13%), but the results did not reach statistical significance because of low power with few events [24]. Future studies will show whether there is a predictive value in the choice of chemotherapy regarding repeated sampling of DTCs during progression of the disease. The persistent presence of DTCs during follow-up was shown to be associated with a significantly increased risk for relapse and death in breast cancer disease in a recently published pooled analysis [25].

The biological tumour characteristics and the clinical stage and follow-up data in the present study are in accordance with those of previous publications in the field [8,9,11,12,26]. The presence of DTCs in 152 of 401 (38%) patients in this study is in line with what previous authors have reported, with a diagnostic rate of 20% to 40% regardless of nodal status [7-9]. Although most authors report a positive association with pathological stage and tumour grade, other investigators have failed to detect any correlation of DTCs with standard prognostic markers [8,9,11,12,27]. The lack of correspondence between accepted prognostic markers and the presence of DTCs in bone marrow spans T1, T2, and T3 disease, and it shows no correlation with nodal involvement [12].

The present study involved a prospective cohort of women diagnosed with early breast cancer from the era of screening mammography. This may have had an effect on the present cohort, which was weighted toward a ‘good’ prognostic profile with a favourable prognosis and few events: small tumours (T1) in 66% of included patients, N0 disease in 61%, and ER-positive tumours in 80%. However, the DTC detection rate is not necessarily dependent on the clinical stage or tumour profile and is still reported to be around 30% to 40% of included patients in recently published studies [8,9,12]. Adjuvant treatment with chemotherapy was given to only 65 of 401 patients. The subgroup analysis of N0 patients in the present cohort gives the impression that DTCs may be of some importance for these patients compared with N+ patients. However, the few events reported to date indicate that the study lacks the power to allow the detection of any significant effect of DTCs in the N0 subgroup. A longer follow-up period will be necessary to fully elucidate this issue.

Standardisation of the detection of DTCs has been widely discussed, and the main limitation of the present study is that it was launched before a standardised method was established. Using antibodies against different CKs to detect epithelial cells in mesenchymal bone marrow is considered to classify these cells as being of tumour origin and thus micrometastases [28]. However, conclusive studies comparing techniques and optimal antibody dilution are not yet available from the same cohort of patients. In the present study, a switch was made from analysing the samples with IF (n = 327) to IC (n = 74), a method with growing acceptance at the time, and with the use of published standards for handling of the samples, enabling a more strict morphological evaluation [18,19]. No differences were found between the subset of patients analysed using the different methods when comparing tumour and patient characteristics or survival. Although there was no statistically significant difference in detection rate, DTCs were diagnosed in 40% of the patients using the IF technique compared with 30% of the patients with IC. We used negative controls for all analysed samples irrespective of the method used. Furthermore, we included bone marrow from 76 healthy adults, and the analyses were positive for epithelial cells in the bone marrow of 19 (25%) of the samples. This illustrates the lack of standardisation of the assays used. Because CK-positive samples were found among the healthy donors in the present study, we cannot exclude the possibility that some of the DTC+ breast cancer patients were incorrectly classified. Although it was not our initial intention, we also tested higher cut-offs for defining DTC positivity without finding any association with prognosis (data not shown).

Not all studies included in the meta-analysis reported whether samples from healthy donors were analysed, and in several of them, control samples were sparse (<50 samples) [7]. Furthermore, false-positive controls have been observed in the use of epithelial-specific markers (CKs), incorrectly classifying haematopoietic cells (HCs) as tumour cells [18,29]. Epithelial-positive rates in bone marrow have been reported in patients without cancer, even after morphological criteria were applied (5% and 30% in two different cohorts) [18,29]. However, the findings in the present study, including 25% positive cases among adult healthy bone marrow donors, raise concerns about the specificity of the method used. One possible mechanism for false-positive staining of HCs in bone marrow is a direct reaction between specific HCs and alkaline phosphatase (AP) [18] using the chromogenic visualisation technique. Staining bone marrow with AP alone gave a strong positive reaction, and further analysis identified these cells as possible plasma cells/pre-B cells [18]. In addition, HCs can express CKs illegitimately and thus stain with anti-CK, but strict morphological evaluation often reveals the characteristics of true DTCs [18]. Morphological evaluation of CK-positive cells is thus crucial for the correct diagnosis of DTCs, and in the present study, we applied the morphological criteria for diagnosis of DTCs analysed by IF and IC [8,18].

A future topic of interest in the field of DTCs is the investigation of the molecular characteristics in individual DTCs with the purpose of finding a marker to monitor treatment susceptibility [8,30]. Molecular characteristics are often diagnosed by the IF-based technique, which is less standardised than the preferred IC-based method. Furthermore, molecular investigation of matched pairs of primary tumours, metastasis, and DTCs may give information about the progression of cancer disease [28]. Of interest is that molecular subclasses of breast cancer tend to express different families of CKs, which can be translated into different detection rates throughout the molecular subclasses when only one antibody against CKs is used [31]. Diagnosis of DTCs during tumour progression must thus consider using multiple antibodies against different CKs to correctly diagnose DTCs with a molecular profile other than that of the primary tumour.

## Conclusions

The present study did not confirm the results of previous publications that suggested that DTCs in bone marrow are an independent prognostic marker of poor prognosis in primary breast cancer. A more standardised detection method of DTC in bone marrow has been proposed since the start of the present study. The invasive nature of the diagnostic procedure of DTCs and technical challenges linked to the method makes the technique unsuitable for inclusion in the standard care of primary breast cancer.

## Abbreviations

AP: Alkaline phosphatase; BCSS: Breast cancer-specific survival; CI: Confidence interval; CK: Cytokeratin; DDFS: Distant disease-free survival; DTC: Disseminated tumour cell; ER: Oestrogen receptor; HC: Haematopoietic cell; HER2: Human epidermal growth factor receptor 2; HR: Hazard ratio; IC: Immunocytochemistry; IF: Immunofluorescence; N+: Metastatic lymph node involvement; N0: No sign of metastatic lymph node involvement; NHG: Nottingham histological grade; PR: Progesterone receptor.

## Competing interests

The authors declare that they have no competing interests.

## Authors’ contributions

AKF was responsible for data acquisition and drafting of the manuscript. POB constructed the database and performed the statistical analysis, CI initiated and designed the study, JI set up the immunofluorescence technique, PEJ was responsible for including patients, PL took part in data acquisition, KL carried out the preparation of the samples and the analysis of them, KR carried out the preparation of the samples and the analysis of them, MF initiated and designed the study, LR was responsible for patients follow-up, data acquisition, survival analysis and drafting of the manuscript. All authors read and approved the final version of the manuscript.

## Funding

The study was supported by funds from the Swedish Cancer Society, Swedish Research Council, Swedish Medical Association, the Gunnar Nilsson Cancer Foundation, the Mrs. Berta Kamprad Foundation, Stig and Ragna Gorthons Stiftelse, Skåne County Council’s Research and Development Foundation and Governmental Funding of Clinical Research within the National Health Service.

## Pre-publication history

The pre-publication history for this paper can be accessed here:

http://www.biomedcentral.com/1471-2407/12/403/prepub
